# Impact of a consumer health information specialization (CHIS) sponsorship program on the ability of public library staff to provide consumer health information

**DOI:** 10.5195/jmla.2021.970

**Published:** 2021-04-01

**Authors:** Elizabeth Kiscaden, Michele Spatz, Susan M. Wolfe, Molly Knapp, Erica Lake

**Affiliations:** 1 elizabeth-kiscaden@uiowa.edu, Greater Midwest Region, National Network of Libraries of Medicine, University of Iowa, Iowa City, IA; 2 mspatz@uw.edu, Pacific Northwest Region, National Network of Libraries of Medicine, University of Washington, Seattle, WA; 3 smwolfe@uw.edu, National Evaluation Office, National Network of Libraries of Medicine, University of Washington, Seattle, WA; 4 molly.knapp@utah.edu, National Training Office, National Network of Libraries of Medicine, University of Utah, Salt Lake City, UT; 5 erica.lake@essentiahealth.org, Research & Evaluation Specialist, Essentia Institute of Rural Health, Essentia Health, Duluth, MN

**Keywords:** consumer health, public libraries, continuing education

## Abstract

**Objective:**

In 2018, the Network of the National Libraries of Medicine (NNLM) launched a sponsorship program to support public library staff in completing the Medical Library Association's Consumer Health Information Specialization (CHIS). The objectives of our study were to: (1) determine whether completion of the sponsored specialization improved ability to provide consumer health information; (2) identify new health information services, programming, and outreach activities at public libraries; (3) investigate benefits of the specialization; and (4) determine the impact of sponsorship on obtaining and continuing the specialization.

**Methods:**

We used REDCap to administer a 16-question survey in August 2019 to 224 public library staff who were sponsored during the first year of the program. We measured competence in providing consumer health information aligned with the eight Core Competencies for Providing Consumer Health Information Services [1] as well as new activities at public libraries, benefits of the specialization to public library staff, career gains, and the likelihood of continuing the specialization based on funding.

**Results:**

More than 80% of 136 participants reported an increase in core consumer health competencies, with a statistically significant improvement in mean competency scores after completing the specialization. Ninety percent of participants have continued their engagement with NNLM, and more than half offered new health information programs and services. While more than half planned to renew the specialization or obtain the Level II specialization, 72% indicated they would not continue without NNLM sponsorship.

**Conclusions:**

Findings indicate that NNLM sponsorship of the CHIS specialization was successful in increasing the capacity of public library staff to provide health information to their communities.

## INTRODUCTION

The Network of the National Libraries of Medicine (NNLM) is the outreach arm of the National Library of Medicine (NLM), one of the 27 institutes, centers, and offices of the National Institutes of Health (NIH). The mission of NNLM is twofold: to “advance the progress of medicine and improve the public health by providing all U.S. health professionals with equal access to biomedical information” and “improve the public's access to information to enable them to make informed decisions about their health” [2]. As such, providing training and continuing education opportunities is integral to supporting the mission of NNLM. On average, NNLM offers 1,500-plus educational activities and connects with more than 50,000 participants each year [3]. NNLM staff develop and present webinars, online courses, and face-to-face training focused on NLM resources, such as PubMed and MedlinePlus, and on NNLM initiatives [4], such HIV/AIDS, data science, and citizen science. NNLM's broad mission means that the learner audience for these offerings includes librarians and library staff, health professionals, researchers, and members of the general public.

In 2017, NLM became a community engagement partner with the *All of Us* Research Program, a part of the NIH Precision Medicine Initiative. The mission of the *All of Us* Research Program is to enroll one million or more participant partners, with an emphasis on populations traditionally underserved in biomedical research, in a longitudinal study to advance precision medicine [5]. As a core component of NLM outreach, NNLM received funding to undertake a three-year pilot to support the *All of Us* Research Program.

A primary goal of the pilot was to increase the capacity of public libraries to provide health information to their communities. In this context, capacity represents public library staff trained to provide health information services and to develop and present health information events, programming, and other activities. As one action to achieve that goal, NNLM launched a national sponsorship program to provide the Consumer Health Information Specialization (CHIS), a specialization offered through the Medical Library Association (MLA) to public library staff.

## Literature Review

The Health and Medicine Division (HMD) of the National Academies of Science, Engineering, and Medicine (NASEM) defines health literacy as “the degree to which individuals have the capacity to obtain, process, and understand basic health information and services needed to make appropriate health decisions [6].” Prior research indicates that those with low health literacy are more likely to be hospitalized, more likely to use emergency services, and less likely to use preventative health care services [7]. This issue is particularly pressing due to the prevalence of low health literacy. According to 2012 data from the Program for the International Assessment of Adult Competencies (PIAAC), only 12% of adults have proficient health literacy skills, a statistic that has changed little over time [8].

A number of studies illustrate the important role that public libraries play in supporting the health of their communities. Libraries in communities of all sizes provide the public with a critical access point to authoritative health information and the necessary technology to locate information online [9]. An important characteristic that makes public libraries ideal partners in public health is their reach—more than 95% of Americans live within a public library service area [10]. Another characteristic is trust: libraries are highly trusted public institutions [11], and more than 70% of Americans believe that libraries help people seeking health information [12].

The U.S. IMPACT Study, developed in partnership with the Institute of Museum and Library Services (IMLS), illustrates how the public applies health information obtained at libraries to make changes to their health behavior [13]. Of the study participants using computers at public libraries, more than a third researched health and wellness issues and roughly half of those reported making follow-up appointments with health care providers [13]. Information obtained about diet and exercise was particularly actionable: of library users researching these topics, more than 80% decided to make changes to their diet or exercise habits [13].

A growing number of public libraries provide health programming in their spaces, often partnering with other community organizations. For example, public libraries have initiated health programming involving pedometers to increase activity [14, 15], after-school nutrition workshops [16], asthma education workshops for children and their caregivers in an inner-city setting [17], healthy cooking demonstrations [19], and digital health literacy training for older adults [19, 20]. Health programming in public libraries has extended to supporting the public in navigating the complex environment of health insurance (such as assistance provided to the public during American Care Act (ACA) enrollment periods [21, 22]), similar to the support offered in libraries for tax preparation.

Previous studies have shown that while public library staff view training to meet the health information needs of their users as a priority, current training opportunities are inadequate. In 2015, a study within 54 branches of the Free Library of Philadelphia was undertaken to investigate how public libraries support population health. Public library staff who participated in the study indicated that their professional training “inadequately prepared” them to serve the health needs of vulnerable populations in their community [23]. Similarly, a survey sent to library directors in the Pennsylvania Library Association revealed that most respondents found their professional training did not prepare them to assist with health issues [24]. Nationally, a survey of public library staff revealed that more than half had not received any training on assisting patrons with health information needs [25].

To support library staff in providing consumer health information, MLA created the Consumer Health Information Specialization (CHIS). The specialization was created with support from NNLM in 2001 to “[offer] training in providing health information services to consumers and recognition for the accomplishment of acquiring new health information skills” [26]. The program offers two levels of specialization, each requiring 12 credit hours of training surrounding eight core competencies [26]. While MLA charges a fee for granting the certificate of completion, participants can obtain all required credit hours by taking advantage of NNLM training opportunities offered at no cost.

Several studies illustrate the success of the specialization program in training public library staff to provide consumer health information to their users. In 2006, the Jewish Healthcare Foundation collaborated with the Carnegie Library of Pittsburgh to create a fellowship for public library staff focused on providing the knowledge and skills necessary for disseminating reliable health information to the public [27]. All 20 participants in the fellowship obtained the CHIS Level I specialization, and outcome measures showed significant improvement in the Fellows’ health information retrieval skills [27].

Another study examined the role of the CHIS in supporting health information knowledge among staff in two public library systems in Oklahoma [28]. Based on interviews with the 38 public library staff participants, it was determined that training made staff more comfortable with health information services and sources [28]. The study concluded with a recommendation for public library staff to pursue the CHIS specialization to meet health information training needs [28].

CHIS was also a cornerstone of the Oklahoma Health Information Specialists Program, launched in 2013 with funding from NNLM [29]. Through this program, 30 public libraries were reached, and 50 public library staff obtained a certificate for Level I or Level II of the specialization [29]. In a follow-up survey of participants, all agreed or strongly agreed that the program improved their knowledge and skills in locating health information [29].

The NNLM *All of Us* National Program goal was to “increase the capacity of public libraries to provide health information to their communities.” To meet this goal, our team at NNLM launched a national sponsorship of the CHIS for public library staff. The sponsorship was open to all U.S. public library staff who were able to complete the 12 credit hours of training required for the specialization. Public library staff completed an online application form, and NNLM paid the $75 specialization fee.

The aim of our study was to determine whether completion of the sponsored specialization was successful in improving the ability of public library staff to provide consumer health information. Our objectives were to (1) measure competence within the 12 competencies of the specialization, (2) identify new health information services, programming, and outreach activities conducted by sponsored staff, (3) investigate the benefits of the specialization to libraries and library staff, and (4) determine the impact of sponsorship on obtaining and continuing the specialization.

## METHODS

We developed a 16-question survey, with questions mapped to each of our objectives (Appendix A). To determine whether the sponsorship program was successful in improving the ability of public library staff to provide consumer health information, we asked participants to rate their competence in the 12 competency areas of the Level I CHIS before and after completing the program[1]. To determine whether the sponsorship program resulted in recipients taking actions, such as offering new health or wellness services, programs, or outreach activities at their libraries, we asked about changes to programming or library services as a result of the specialization training. Finally, to assess the value of the specialization to recipients and their libraries, we asked open-ended questions about the benefits and expectations of the program. Respondents were also asked about their willingness to pay out of pocket for the renewal of their Level I specialization or application for the Level II specialization. The purpose of this question was to determine whether the sponsorship was successful in removing financial barriers to obtaining the certificate.

We sent survey invitations to a total of 224 sponsored public library staff who completed either the Level I or Level II CHIS between April 2018 and April 2019, the first year of the sponsorship program. While sponsorship was open to all U.S. librarians and library students, those invited to participate in the survey were limited to recipients who indicated on their original sponsorship application that they were employed at public libraries at the time of receiving sponsorship. The survey was distributed in August 2019, and respondents were given three weeks to complete the survey, with a final reminder email sent to those who had not responded before the due date.

We collected and managed study data using REDCap, a secure, web-based software platform designed to support data capture for research studies hosted at the University of Washington [30, 31]. One team member analyzed qualitative data by reviewing responses to identify themes and coding each statement by theme as either 1 (theme present) or 0 (theme not present), allowing for quantification of themes. Stata statistical analysis software [32] was used to create variables and clean and analyze quantitative data.

To evaluate the effect of specialization completion on participants’ ability to provide consumer health information, we created mean pre-test and post-test scores for each CHIS competency and a total mean competency score. We then used paired t-tests to test differences between the retrospective pre-test and post-test scores to determine whether there was a change in ratings of competence before and after completing the specialization. We evaluated internal consistency using Cronbach's alpha (α=.92 and .92, respectively). The 12 questions used to assess knowledge and skills on the pre-test and post-test were derived directly from the CHIS competencies. Respondents rated themselves on a Likert scale: (1) No knowledge, (2) Beginner (some experience or basic knowledge, (3) Proficient (satisfactory level), (4) Advanced (better than most), or (5) Expert (superior level of skill).

## RESULTS

Overall, 136 sponsorship recipients (61%) responded to the survey, with the number of responses ranging from 115 to 136 for each question. Of the 136 respondents, 128 (94%) were still employed at public libraries; the remaining 8 (6%) had taken positions at academic or special libraries. Given that all respondents were working at public libraries when they obtained training and applied for the specialization, these responses were retained in the data.

### Competence in consumer health information

**Figure 1:**
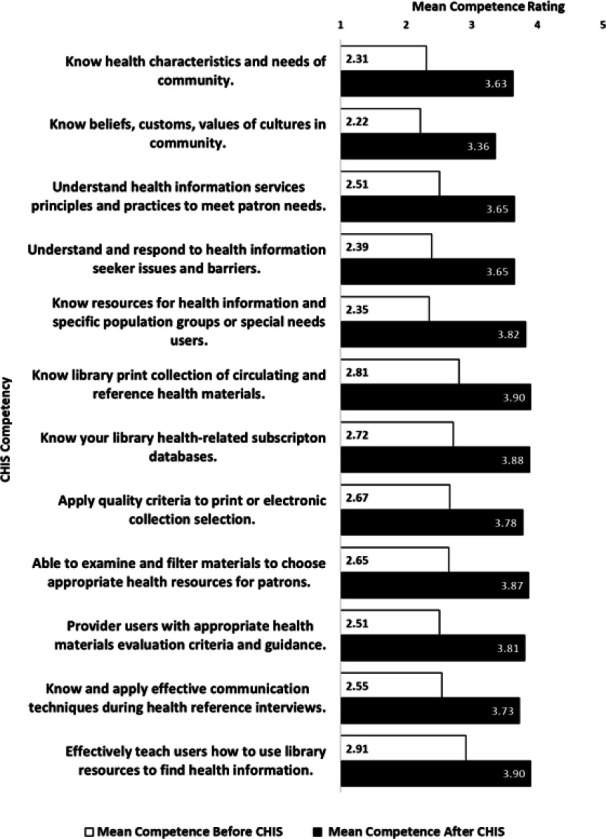
Mean competence before and after completion of CHIS

The ability to provide consumer health information was measured by self-reported competence for each of the CHIS competencies before and after completing the specialization. Participants responded to a Likert scale ranging from 1 (No knowledge) to 5 (Expert) (see Figure 1). On average, respondents reported statistically significant increases in competence for each of the topics listed in the survey. Mean competency in each of the 12 areas before specialization ranged from 2.23 (Beginner) to 2.91 (Proficient), while mean competency after completion ranged from 3.35 (Proficient) to 3.89 (Advanced). A t-test comparing the mean self-reported total competency score before (M=2.6, SD=.64) and after completion of the specialization (M=3.7, SD=.51) indicated that respondents reported significantly higher competence after completing the specialization (t(133) = 23.9, p<.05).

### New consumer health activities at public libraries

**Figure 2:**
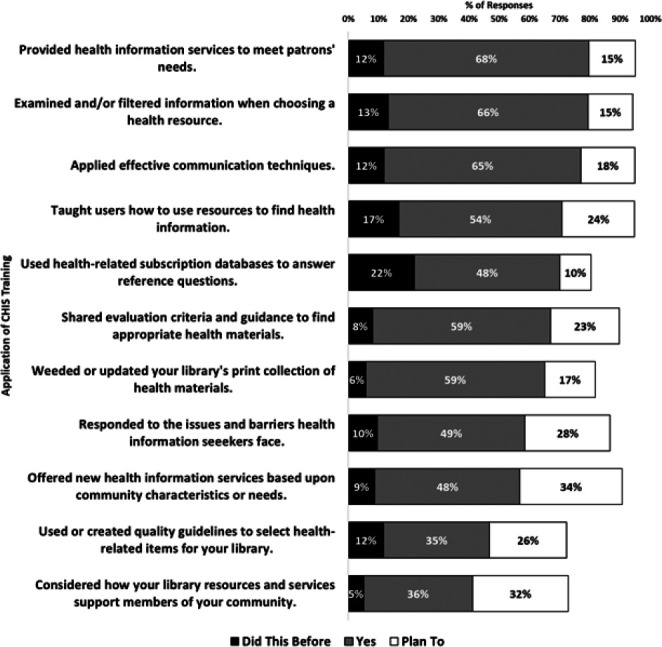
Current and planned application of CHIS training

To measure whether the sponsored specialization resulted in new services, programs, or outreach activities at public libraries, survey respondents were asked how they used or plan to use their training related to 11 different areas of library services (Figure 2). Of 133 participants who responded to this question, 93% (n=124) have used the training they received in at least one new way and 79% (n=107) planned to do so.

**Figure 3:**
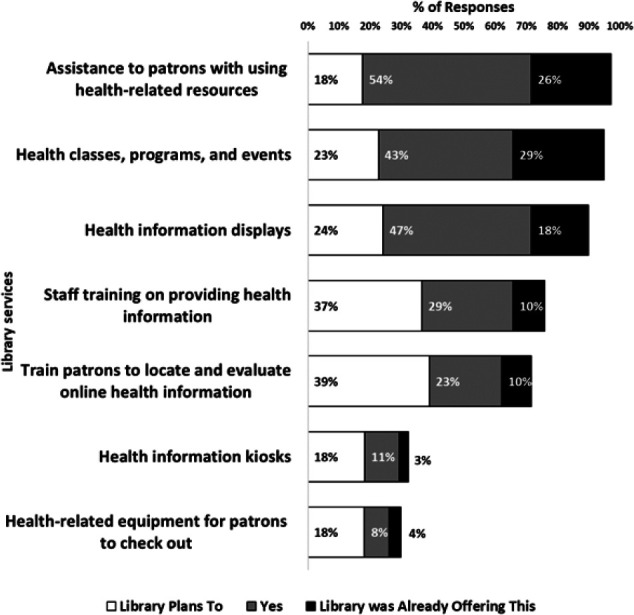
Current or planned changes to library services after CHIS

Participants were asked how the services at their library changed after they completed CHIS (Figure 3), and 134 participants (99%) responded. Roughly four in five respondents (n=110) reported that their libraries are now offering at least one new health-related service, and an additional 12 percent (n=16) plan to offer at least one new health-related service in the future.

To explore this in more depth, survey respondents were asked an open-ended question about how their library benefited. Among the 99 respondents who answered, the most frequently mentioned benefits were the following: improved existing staff skills, including training of staff by the sponsorship recipient (39%; n=39); improved existing services (37%; n=37); fulfillment of a community need (18%; n=18); launch of new services or programs (16%; n=16); and the availability of the staff member with the specialization to serve as an overall resource to the library (14%; n=14). Other benefits included making new products or resources available, increased staff confidence to provide health information and programming, increased patron or community confidence in the library as a health resource, awareness of funding opportunities, and improved outreach.

Nearly all respondents (98%) agreed or strongly agreed that the sponsored specialization increased their confidence to offer health information and other services, including answering health reference questions (98%), teaching patrons to locate health information (96%; n=129), and offering health information services within their organizations (94%; n=126). Additionally, most respondents (79%; n=106) reported that obtaining the specialization motivated them to reach out to new groups of potential library users.

### Benefits to public library staff

At the time of the survey, most respondents did not report they had benefited professionally from obtaining the certificate. Only 14% (n=19) reported changes in their library position or career, promotions, expanded duties, and other gains. One respondent shared that they obtained a new job when the library that hired them planned to implement a consumer health information corner.

Regarding continued engagement with NNLM, the CHIS, and consumer health information, 90% (n=121) of the 134 respondents have taken additional actions. Seventy nine percent (n=106) of participants recommended the specialization program, NNLM programs and services, or consumer health activities to other library staff. More than half of respondents (55%; n=74) advocated for library staff at their institution to provide consumer health information, and 38% (n=51) have taken more classes to strengthen their consumer health information knowledge. More than a third of respondents (33%; n=44) interacted with their NNLM regional medical library, and 19% (n=25) applied for funding from their NNLM regional medical library. For each of these, most respondents who had not taken these actions indicated that they plan to do so.

### Impact of sponsorship

When asked if obtaining the specialization met their expectations, all 136 respondents reported that it did. When asked how it met their expectations, respondents indicated that the specialization made it possible for them to obtain new knowledge about health information (23%; n=31), taught them about new resources (14%; n=19), increased their confidence to provide health information (13%; n=18), and increased their skills (10%; n=15). Although the specialization met expectations, respondents’ intentions to renew their Level I specialization or continue to Level II without the support of the NNLM sponsorship program was low. While more than half of respondents (65%; n=87) planned to renew their CHIS certificate, most (72%; n=96) would not pay out of pocket or were unsure about doing so. Similarly, a third (35%; n=47) planned to obtain Level II CHIS, but most (66%; n=88) of these participants were unsure about paying out of pocket or would not do so.

## DISCUSSION

The purpose of the sponsorship pilot undertaken by NNLM was to support a key goal of the NNLM *All of Us* National Program—namely, to increase the capacity of public libraries to provide health information to their communities. Our findings indicate that the sponsorship pilot was successful in supporting this goal by increasing the skills, knowledge, and confidence of participants in providing consumer health information. This was illustrated by the increase in mean self-reported competency for each of the CHIS competencies and the increase in confidence after completing the specialization.

Training opportunities offered toward obtaining the specialization, which NNLM makes available at no cost, address a need for professional development communicated by staff in prior studies [23, 24, 25]. Further, the skills and confidence gained by public library staff through the specialization training establish expertise at public libraries across the country. As one responded shared, “there is now a point person for health-related questions, which is an area that frightens most librarians.” This underscores the value of having a library team member trained in providing health information.

The pilot was successful in generating new services, programming, and outreach activities at public libraries. These activities further establish public libraries as a resource for health information and wellness activities within their communities. Examples shared by respondents included revamping their library's collection of health information databases, establishing a consumer health corner in the library, programming a guest speaker on a health topic, bringing in a mental health first aid class for staff, creating a health information page on the library's website, and initiating a grant-funded, district-wide health and wellness initiative. One respondent shared that obtaining the specialization “opens the doors to programming opportunities we hadn’t thought of before or didn’t want to do for fear of providing misinformation.”

Beyond supporting the goal of the NNLM *All of Us* National Program, the training and outreach activities initiated by the pilot support the mission of NNLM to “[improve] the public's access to information and enable them to make informed decisions about their health” [3]. Many sponsorship recipients interacted for the first time with their Regional Medical Library after completing the specialization, which was perhaps not surprising given that the training and sponsorship raised awareness of NNLM. Also, the sponsorship program resulted in an increase in new NNLM public library funding applicants, as demonstrated by the number of survey respondents who later applied for funding. If NNLM seeks new funding applicants from public libraries, continuing the sponsorship is proven to generate these applications.

NNLM and the Medical Library Association should take note of our findings that the specialization met the expectations of participants. Sponsored participants shared many positive comments about the specialization, such as, “I believe the certificate was well worth the time investment.” Another shared, “It's exceeded my expectations. I learned more than I expected and I continue to learn through the amazing outreach for the NNLM.” Pairing the training opportunities offered by NNLM with the MLA CHIS appears to be a successful combination. Given the support for the specialization from respondents in this study, MLA may want to consider more actively marketing to public library audiences.

More than half of our respondents were motivated to continue the specialization and complete Level II, but some found obtaining the specialization to be more of an incentive than others. Some became advocates for the specialization; as one respondent shared, “I am advocating for every branch to have at least one CHIS-certified staff member and for all of our reference staff at our main library to obtain CHIS certification as well.” In contrast, a respondent who did not intend to continue the specialization shared, “I really attended the training for the sake of knowledge. There is no tangible benefit to maintaining the actual certificate.” If the specialization is to be advanced or maintained by public library staff, the value must be made clear. A collaboration with state public library accreditation programs may add value to the specialization, given that the training would count toward required certification also.

This study illustrates the financial barrier to continuing the specialization, as most respondents did not plan to continue without sponsorship. For NNLM and the NNLM *All of Us* National Program, this clearly demonstrates a need to continue the national sponsorship so as to meet the goal of increasing the capacity of public libraries to provide health information to their communities. Given the gains in competence and confidence in providing health information to patrons revealed by our study, public libraries can justify investing in the specialization for staff members. The training is provided at no cost by NNLM, the specialization fee is modest, and it offers a high return on investment.

One limitation of this research is the risk of self-selection bias, as the assessment was sent only to recipients of the NNLM sponsorship for the specialization. Contact information used to administer the survey was collected when individuals applied for sponsorship. Missing from the results are responses from those who may have attempted the process and did not follow through with obtaining sponsorship. Consequently, results may be positively skewed toward those who were successful in obtaining the specialization.

Another limitation is data from those respondents who had exited public libraries between the receiving sponsorship for the specialization and completing the survey. While these respondents were employed in a public library when they achieved the specialization, their change in employment may have affected their ability to stage health information events, programming, and other activities in their libraries. Additionally, the survey received responses from just 61% of the sponsorship recipients contacted, and no survey questions had a 100% completion rate.

The NNLM national sponsorship pilot of the CHIS was undertaken to meet a goal of the NNLM *All of Us* National Program to increase the capacity of public libraries to provide health information to their communities. Our research shows that sponsorship was successful in meeting this objective. We found that most public library staff found value in the specialization, but many would not continue the specialization without financial support.

## Data Availability

Data associated with this article are available in The Hive: the University of Utah Research Data Repository at https://doi.org/10.7278/S50D1DAY2QQQ.

## SUPPLEMENTAL FILES

**Appendix A:** CHIS Sponsorship Assessment
