# Supplementary material for: Impact of a consumer health information specialization (CHIS) sponsorship program on the ability of public library staff to provide consumer health information
Source: J Med Libr Assoc. 2021 Apr 1;109(2):267–74. doi: 10.5195/jmla.2021.970 (PMC8270365; doi:10.5195/jmla.2021.970)
Supplement: Supplementary file 1 — Appendix A: CHIS Sponsorship Assessment [file jmla-109-2-267-s01.pdf]

CHIS Sponsorship Assessment

Please complete the survey below.

Thank you!

Zip code

____
(Please enter your 5-digit zip code.)

In which type of library do you work?

- ☐ Academic Health Sciences
- ☐ Academic Non-Health Sciences
- ☐ Corporate/Industry
- ☐ Government
- ☐ Hospital
- ☐ K-12
- ☐ Public
- ☐ Other

(Please check all that apply.)

If other, please specify:

____
(other_library)

What motivated you to earn the Consumer Health Information Specialization (CHIS)?

____

Please rate your competence for each of the following areas BEFORE you began working towards earning the Consumer Health Information Specialization (CHIS).

	No Knowledge	Beginner (Some experience or basic knowledge)	Proficient (Satisfactory level)	Advanced (Better than most)	Expert (Superior level of skill)
Know the health characteristics and health needs of the community served by your library.	○	○	○	○	○
Know the beliefs, customs, and values of the different cultures in your community, and how they can influence thoughts and actions around health, illness, and health care decisions.	○	○	○	○	○
Understand the principles and practices related to providing appropriate, relevant health information services to meet the needs of different types of patrons.	○	○	○	○	○

Understand and respond to the issues and barriers faced by health information seekers.	○	○	○	○	○
Know the major resources for general and specialized health information, as well as resources for specific population groups or for users with special needs.	○	○	○	○	○
Know your library's print collection of circulating and reference health materials.	○	○	○	○	○
Know your library health-related subscription databases.	○	○	○	○	○
Apply quality criteria when selecting items for the print or electronic collection.	○	○	○	○	○
Have the ability to critically examine and filter materials when choosing an appropriate health resource for a particular patron.	○	○	○	○	○
Provide users with evaluation criteria and guidance for finding appropriate health materials.	○	○	○	○	○
Know and apply effective communication techniques during health reference interviews.	○	○	○	○	○
Effectively teach users how to use search engines, library catalogs, subscription databases, reference materials, and online resources to find health information.	○	○	○	○	○

Please rate your competence for each of the following areas AFTER you began working towards earning the Consumer Health Information Specialization (CHIS).

	No Knowledge	Beginner (Some experience or basic knowledge)	Proficient (Satisfactory level)	Advanced (Better than most)	Expert (Superior level of skill)
Know the health characteristics and health needs of the community served by your library.	○	○	○	○	○

Know the beliefs, customs, and values of the different cultures in your community, and how they can influence thoughts and actions around health, illness, and health care decisions.	○	○	○	○	○
Understand the principles and practices related to providing appropriate, relevant health information services to meet the needs of different types of patrons.	○	○	○	○	○
Understand and respond to the issues and barriers faced by health information seekers.	○	○	○	○	○
Know the major resources for general and specialized health information, as well as resources for specific population groups or for users with special needs.	○	○	○	○	○
Know your library's print collection of circulating and reference health materials.	○	○	○	○	○
Know your library health-related subscription databases.	○	○	○	○	○
Apply quality criteria when selecting items for the print or electronic collection.	○	○	○	○	○
Have the ability to critically examine and filter materials when choosing an appropriate health resource for a particular patron.	○	○	○	○	○
Provide users with evaluation criteria and guidance for finding appropriate health materials.	○	○	○	○	○
Know and apply effective communication techniques during health reference interviews.	○	○	○	○	○

Effectively teach users how to use search engines, library catalogs, subscription databases, reference materials, and online resources to find health information.	○	○	○	○	○

Have you used the training you received in obtaining your certificate to do the following:

	Yes	I plan to	No	I did this before obtaining CHIS	Does not apply
Offered new health information programs or services based upon the health characteristics and health needs of your community.	○	○	○	○	○
Explored the health beliefs, customs, and values of a different culture in your community, and considered how your library resources and services support them.	○	○	○	○	○
Provided appropriate, relevant health information services to meet the needs of different types of patrons.	○	○	○	○	○
Responded to the issues and barriers faced by health information seekers.	○	○	○	○	○
Weeded or updated your library's print collection of circulating and reference health materials.	○	○	○	○	○
Used your library's health-related subscription databases to answer reference questions.	○	○	○	○	○
Used or created quality guidelines to select health-related items for your library's print or electronic collections.	○	○	○	○	○
Critically examined and/or filtered information when choosing an appropriate health resource for a particular patron.	○	○	○	○	○

Shared evaluation criteria and guidance for finding appropriate health materials with library users.	○	○	○	○	○
Applied effective communication techniques during a health reference interview.	○	○	○	○	○
Taught users how to use search engines, library catalogs, subscription databases, reference materials, and online resources to find health information.	○	○	○	○	○

As a result of obtaining your certificate, please tell us if you are now offering any of the following at your library:

	Yes	Library plans to	No	Library was already offering this
Health classes, programs, and events	○	○	○	○
Health information displays	○	○	○	○
Health information kiosks	○	○	○	○
Health-related equipment for patrons to check out (for example, blood pressure machines or exercise equipment)	○	○	○	○
Assistance to patrons with using health-related resources (for example, MedlinePlus or PubMed)	○	○	○	○
Staff training on providing health information	○	○	○	○
Patron training on locating and evaluating online health information	○	○	○	○

How much do you agree with each of the following statements regarding the class(es) you took to obtain your certificate? Taking the classes:

	Strongly agree	Agree	Neither agree nor disagree	Disagree	Strongly disagree
Increased my confidence to answer health reference questions more efficiently or effectively.	○	○	○	○	○

Increased my confidence in teaching patrons to locate health information questions more efficiently or effectively.	○	○	○	○	○
Increased my confidence to offer health information services within my organization.	○	○	○	○	○
Motivated me to reach out to new groups of potential library users.	○	○	○	○	○

Did obtaining your certificate result in any changes in your library position or career? ○ Yes ○ No

If yes, how have you benefited?

- ☐ Received a pay raise
- ☐ Received a promotion
- ☐ Expanded my duties
- ☐ Other (please describe box)

(Please check all that apply.)

If other, please specify: ________________________________

How does having your CHIS certificate benefit your library? ________________________________

Please check the answer that describes whether you have done the following as a result of receiving your CHIS:

	Yes	I plan to	No	I did this before obtaining CHIS	Does not apply
Taken more classes to strengthen your consumer health information knowledge.	○	○	○	○	○
Recommended CHIS training, resources, NNLM programs, and services or consumer health activities to other library staff.	○	○	○	○	○
Interacted with your NNLM regional medical library.	○	○	○	○	○
Applied for funding from your NNLM regional medical library.	○	○	○	○	○
Advocated for library staff at your institution to provide consumer health information.	○	○	○	○	○

Has obtaining the certificate met your expectations?

○ Yes
○ No
(expect)

If yes, please tell us how:	______________________ (yes)
If no, please tell us why:	______________________ (expect_no)
Do you plan to renew your certificate?	◯ Yes<br>◯ No<br>◯ Unsure<br>(renew)
If you had to pay out of pocket ($75) to renew Level I of the CHIS certificate, would you do so?	◯ Yes<br>◯ No<br>◯ Unsure<br>(renew_level1)
Do you plan to obtain the Level II CHIS certificate?	◯ Yes<br>◯ No<br>◯ Unsure<br>(level_II)
If you had to pay out of pocket ($75) to obtain Level II of the CHIS certificate, would you do so?	◯ Yes<br>◯ No<br>◯ Unsure<br>(level_ii_pay)
If there is anything else you would like to share about the CHIS certification process or your experience with CHIS, please share it below.	______________________ (share)
